# Aim high, hit ×10: psychological strategies driving the success of South Korean archers

**DOI:** 10.3389/fpsyg.2024.1482897

**Published:** 2025-01-29

**Authors:** Joonyoung Lee, Suyoung Hwang

**Affiliations:** ^1^Department of Health, Physical Education, and Recreation, Jackson State University, Jackson, MS, United States; ^2^Research Institute of Exercise Rehabilitation and Convergence, Gachon University, Incheon, Republic of Korea

**Keywords:** Korean archer, peak performance, mental strategies, ×10, content analysis, coping mechanism, elite athlete performance, sport psychology

## Abstract

**Introduction:**

South Korean archers have excelled in global competitions. However, limited research exists on the factors contributing to their long-term success, particularly in the context of achieving ×10 scores. This qualitative study investigated the exceptional success of South Korean archers, focusing on their achievement of ×10 scores. Using a content analysis approach guided by epistemological perspectives, we examined the techniques, mental strategies, and coping skills for environmental factors influencing ×10 shooting performance.

**Methods:**

Data were collected using convenience and snowball sampling from 65 Korean archers (*M*_age_ = 18 years ± 0.5; 55% female and 45% male) with experience hitting ×10 scores. A hybrid coding approach, combining deductive and inductive methods, was used to analyze responses. Deductive analysis applied an 8-step archery model, while inductive coding identified emergent themes. Trustworthiness was ensured through multiple rounds of coding, expert validation, and member checking.

**Results:**

The findings revealed several key themes across four phases of archery performance. In the preparation phase, confidence and positive mindset (30.9%), loss of self-consciousness (21%), mastery-oriented goals (18.5%), performance-oriented goals (18.5%), and psychological regulation strategies (11.1%) were significant. During the drawing and anchoring stages, physical techniques and stability (55.4%), surrounding environments (35%), and attentional focus (9.6%) were crucial. In the aiming and release phase, wind management strategies (48.4%), natural posture and kinesthetic balance (35.7%), and confidence and positive outcome expectation (15.9%) were prominent. Lastly, in the post-shot phase, positive emotion elevation (74%), cognitive affirmation (14%), and tension management (12%) were key themes. These themes illustrate the comprehensive strategies, techniques, and coping skills essential for achieving ×10 scores in archery.

**Conclusions:**

The holistic approach equips Korean archers to manage psychological demands and maintain consistent performance under pressure. The findings provide practical applications for archers, coaches, and sport psychologists, guiding the development of interventions to enhance mental strategies, physical techniques, and environmental coping skills, thereby improving performance outcomes in archery sports.

## 1 Introduction

For the past 40 years, South Korean Olympic archers have won approximately 60% of the possible gold medals−27 out of 45—since their debut in 1984 (Kim, 2023). This high level of performance extends the World Archery Championships and the Asian Games (Choi and Ok, 2016; Kim et al., 2015). South Korean's male and female national teams hold top positions in the recurve bow category, which requires significant physical strength and skill (World Archery, 2023). Such long-term success raises questions about the factors contributing to Korean archery's strength. Historical and cultural elements, like the use of metal chopsticks to enhance fine motor skills, and robust support from the government and industry, further bolster this strength (Choi and Ok, 2016; Han, 2008; Huh, 2016).

Unique training methods in Korean archery, such as field-oriented training, simulated competition environments, and mental resilience exercises, play a crucial role (Park et al., 2016). These programs, emphasizing mental and psychological skills, are vital for a sport that relies heavily on mental fortitude (Kim et al., 2021). Archery demands acute focus, emotional regulation, imagery, and rapid decision-making, essential for success in high-stakes competitions (Kim et al., 2015; Kim and Oh, 2017; Kim and Kim, 2020; Medeiros Filho et al., 2008). Through a combination of technical and mental preparations, archers are equipped to deliver optimal performance in competitions (Robazza and Bortoli, 1998; Salleh et al., 2020).

**Figure 1:**
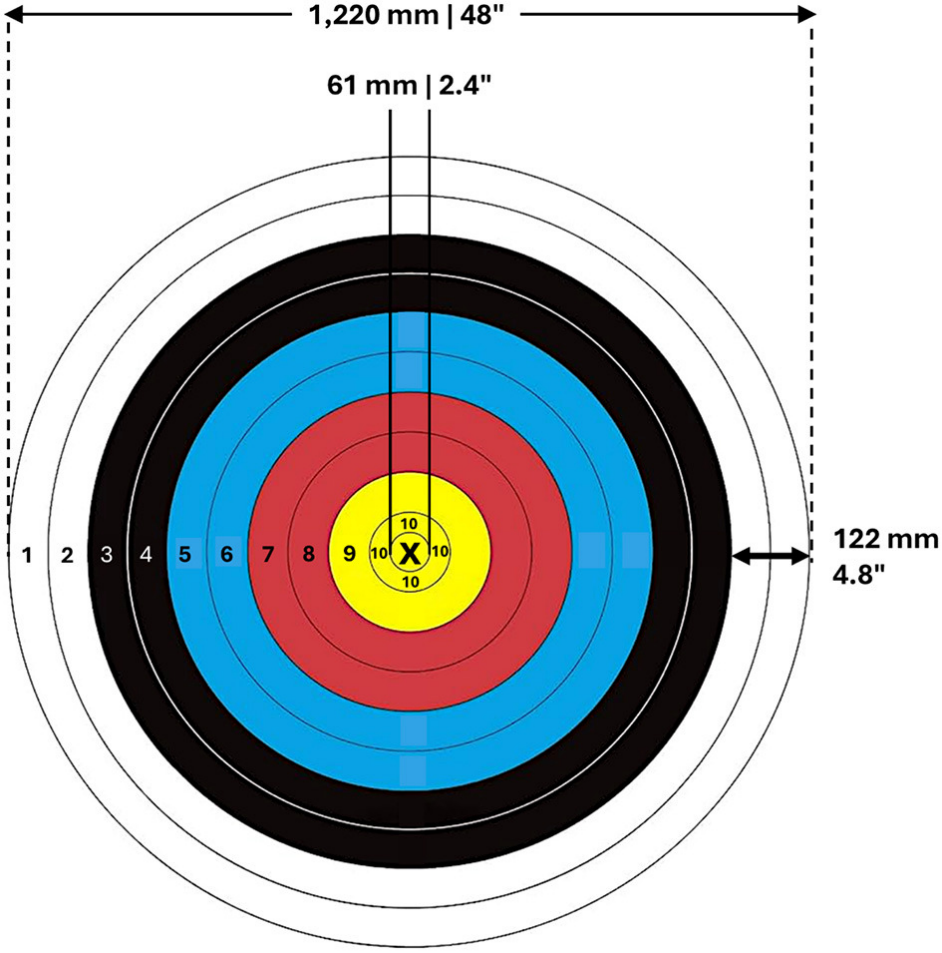
Diagram of archery target with ×10 ring and diameter measurements. The ×10 ring is half the diameter of the 10-ring, and both yield the same score of 10 points.

Despite evolving rules and fluctuating conditions, one fundamental principle in archery remains unchanged: the archer who consistently hits the center of the target is the winner. Achieving such consistency requires extraordinary skill, endurance, and mental resilience. In the preliminary round, archers shoot 72 arrows at a target 70 m away, with their total score determining their ranking. Based on these rankings, archers advance to elimination rounds, where each set consists of three arrows, and the first to reach six set points wins. Matches range from 9 to 15 arrows (40 seconds per arrow), with a single-arrow shoot-off used to break ties after five sets. This format tests both precision and mental focus, particularly given the challenge of hitting a 1220 mm (48^′′^) target face at 70 m (World Archery, 2024). The 10-ring measures 122 mm (4.8^′′^), while the × -ring, at half its diameter, is only 61 mm (2.4^′′^) (see Figure 1). In addition to the technical demands, archers must navigate environmental factors such as wind speed, atmospheric conditions, and ambient noise, which can further challenge their consistency and performance (Kolayis et al., 2014; Venkat, 2021).

Within this context, archers aim to achieve the pinnacle of performance indicators in archery, known as the “ ×10” score. The term ×10 refers to an ideal score of 10 points, denoting both accuracy and precision, commonly referred to as a bullseye or × -ring (Goldblatt and Acton, 2012; Haywood and Catherine, 2013). Achieving an ×10 score is a considerable feat, requiring an elevated degree of skill, consistency, and precision in one's archery technique. The probability of hitting the ×10 score is relatively low, even among professional archers, due to the ring's small diameter of about 61 mm (2.4'') and the numerous variables that can influence an arrow's flight. When two archers have tied total scores, the number of ×10 hits serve as the tiebreaker, highlighting the score's significance in determining match outcomes (Haywood and Catherine, 2013). As a result, the ability to consistently achieve ×10 scores is crucial for winning.

While technical (e.g., stance, grip, and release methods; Stone, 2007; Vendrame et al., 2022), psychological (e.g., confidence, focus, visualization, goal setting, and positive self-talk; Haywood, 2005; Kim et al., 2021; Robazza and Bortoli, 1998; Salleh et al., 2020) and environmental (e.g., weather conditions, wind, noise, and the presence of an audience; Kim et al., 2015; Lu et al., 2021; Vrbik et al., 2021) factors are known to influence archery performance, the exact mechanisms enabling archers to hit the ×10 target are not well understood. The success of Korean archers is often attributed to their rigorous training (Park et al., 2018, 2016). However, without a detailed understanding of the comprehensive mechanisms underpinning this success, coaches and athletes in other regions may struggle to replicate these results. A thorough examination of the shooting procedure is necessary to comprehend the mechanisms that enable consistent targeting of the ×10.

In particular, the specific procedural dimensions—ranging from preparation to post-shot phases, including detailed strategies, techniques, and coping skills for external factors—that contribute to this success have not been fully explored. Although one previous study (Kim et al., 2015) investigated the comprehensive factors (i.e., fitness, skill, and mental) influencing athletic performance among Korean archery experts (i.e., professors and coaches specializing in archery) and amateur archers, it did not focus on achieving the ×10 score or best performance. Instead, Kim et al. (2015) defined archery performance as “comprehensive competition ability.” This gap in understanding can hinder the development of optimized training programs and coaching strategies. In our study, we explored the specific procedural dimensions based on the archery sequence of movements, elaborating eight-step process: (1) stance, (2) nocking the arrow, (3) hooking and gripping, (4) pre draw, (5) full draw, (6) aiming and expansion, (7) release, and (8) follow-through (Yi et al., 2007). By doing so, this study can identify specific factors within these sequences and provide a comprehensive overview of practical applications that benefit archers globally.

As global interest in the factors contributing to the long-term success of Korean archers increases, the potential beneficiaries of this study's findings are substantial. Therefore, this study aimed to explore the detailed strategies, techniques, and coping skills for external factors that enable Korean archers to hit the ×10, focusing on the sequences of archery movements. By mapping these elements comprehensively, the research sought to provide valuable insights into how archers manage internal and external factors to optimize their performance. The research questions guiding this study were:

(i) What strategies, techniques, and coping skills for external factors do Korean archers employ to hit ×10 scores?

(ii) How do the contributing factors to hitting ×10 scores relate to the sequential phases of archery movement?

## 2 Methods

### 2.1 Content analysis approach

We employed a conceptual content analysis (Krippendorff, 2018), guided by an epistemological perspectives (Gray, 2021), to explore experiences and identify the key strategies employed by Korean archers to achieve consistent high performance, particularly in hitting the ×10 target. This method involves systematically categorizing textual data to quantify the presence of specific concepts within the data set (Elo et al., 2014; Krippendorff, 2018). By converting qualitative data into quantitative measures, the content analysis provided a clear and objective way to determine the prevalence of specific strategies and techniques among Korean archers. This manuscript complies with the APA Style Journal Article Reporting Standards for Qualitative Research (Levitt et al., 2018).

### 2.2 Participants and procedures

Upon receiving approval from the Institutional Review Board (IRB) of the corresponding author's institution, data were collected using a combination of convenience sampling and snowball sampling methods (Palinkas et al., 2015). Participants were initially identified from a pool of athletic specialists and members of professional teams registered with the Korean Sport and Olympic Committee in 2023. Participants were selected based on their competitive experience and consistent high-level performance in archery, with an average score of 327 out of 360 in 70-m matches and experience achieving at least one ×10 score. With the assistance of a colleague who had previously been a member of a professional archery team and still maintains connections with multiple archery teams across South Korea, we disseminated recruitment flyers to coaches and athletes at six universities with established archery teams as well as to members of 12 professional archery teams within the nation. Questionnaire packages were subsequently distributed to athletes and archery teams who consented to participate in the study. We requested that coaches or supervisors return the packages only from athletes who had experienced shooting a ×10 score. Furthermore, the questionnaire included an item specifically asking about their experience of achieving a ×10.

Our final sample included a total of 65 archers (*M*_age_ = 18 years ± 0.5; 55% female and 45% male), which is considered sufficient to reach data saturation (>20 samples; Hagaman and Wutich, 2017). The participants had an average archery career duration of 8 years. Throughout their athletic careers, they achieved an average of 4.5 times of ×10 hits. All participants voluntarily consented to participate in the study.

The open-ended survey was designed to capture archers' past experiences and the factors contributing to their successful shots for a ×10 (Table 1). The items included targeted inquiries focusing on strategies, techniques, and coping skills for external factors, mindset, and feelings (e.g., mental and emotional states, physical conditions, sensations, and environmental factors). These inquiries were aligned with the sequential steps involved in an archer's arrow-shooting techniques (Yi et al., 2007). Specifically, the questionnaires were distributed to 12 expert panels (e.g., one national team coach, five national team archers, and six former players) to verify the validity and relevance of our open-ended questions. Throughout this process, we refined the terminology and statements commonly used in archery to ensure the questions were easily understandable for the archers.

**Table 1:** Sample open-ended questions.

1.	Please provide your demographic information. a) Age: b) Gender: c) Archery career duration: d) Average score in matches at a distance of 70 meters: e) Number of times you have hit the ×10 target during your archery career:
2.	Can you describe in detail your experiences of shooting ×10 scores, including the strategies, techniques, coping skills, mindset, and feelings based on the following stages? Please provide specific examples and elaborate on the thoughts and actions that helped you succeed in each stage. a) Stance b) Nocking the arrow c) Hooking and gripping d) Pre draw: e) Full draw: f) Aiming and expansion: g) Release: h) Follow-through:
3.	When you shot a ×10, were there specific words or phrases that came to mind during each of the following stages? Please elaborate on any self-talk, motivational phrases, or mental cues that helped you perform successfully. a) Stance: b) Nocking the arrow: c) Hooking and gripping: d) Pre draw: e) Full draw: f) Aiming and expansion: g) Release: h) Follow-through:

The open-ended survey allowed participants to express their experiences in their own words, leading to rich, qualitative data regarding hitting the ×10 target. The participants' responses were entered into Microsoft Excel to facilitate both organizational efforts and preliminary data exploration.

### 2.3 Data analysis

**Figure 2:**
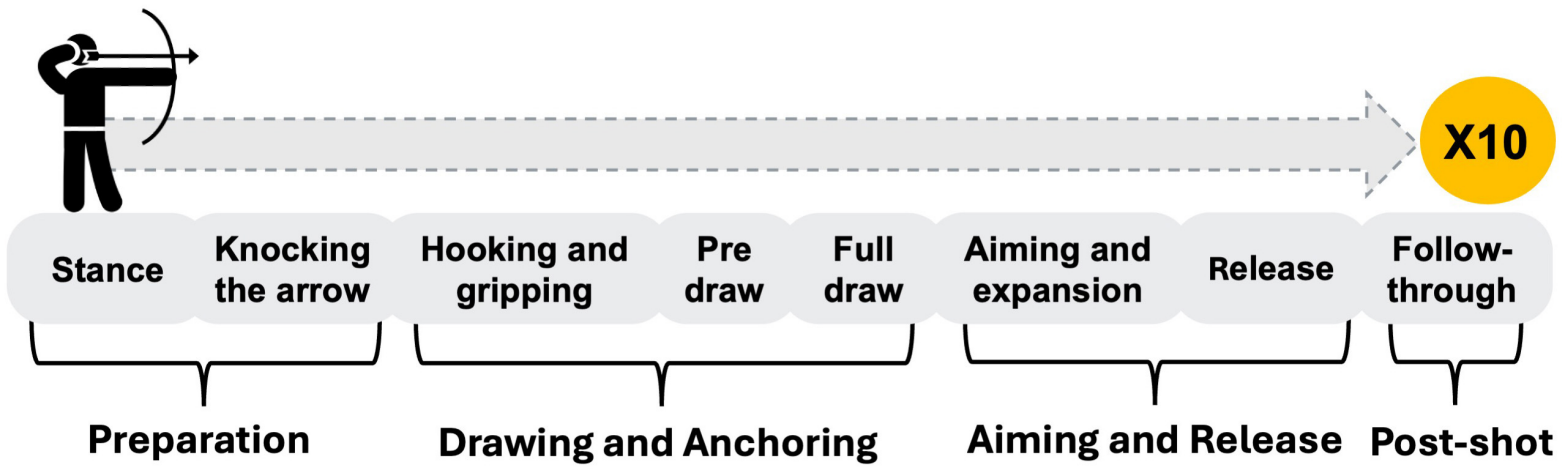
Phases of archery movement steps for achieving a ×10 score. The movement steps are categorized into four phases: preparation, drawing and anchoring, aiming and release, and post-shot.

We employed a hybrid coding approach, incorporating both deductive and inductive methods, to leverage the richness of the data and produce more transparent and relevant findings (Krippendorff, 2018). In the initial phase, we employed a deductive approach by applying the 8-steps of archery performance movement model (Yi et al., 2007) to frame our analysis. This provided a structured lens through which we analyzed the responses, focusing on how each step contributed to achieving a ×10 score. After discussion and feedback from experts in archery, we grouped the movement steps into four broader phases: (1) preparation (stance; nocking the arrow), (2) drawing and anchoring (hooking and gripping; pre draw; full draw), (3) aiming and release (aiming and expansion; release), and (4) post-shot (follow-through) (see Figure 2).

We then moved to an inductive approach, creating a codebook to ensure consistency and clarity in the coding process. Both researchers carefully read through all the responses to deeply understand the archers' past experiences, allowing new themes to emerge naturally from the data. Through open coding, we identified recurring themes related to factors like strategies, techniques, and coping skills for environmental factors. Finally, we refined our main themes until we all agreed on the findings. The coding process was continually reviewed by a colleague who is an expert in qualitative scholarly works and archery sports.

To enhance the trustworthiness of this study, we implemented a rigorous coding process for the qualitative data (Shenton, 2004). This involved multiple rounds of independent coding, followed by discussions to reach consensus. Furthermore, we sought external validation by consulting with an expert in the fields of archery and sport psychology to review our findings (Elo et al., 2014).

### 2.4 Researcher positionality

Both authors of this research are former athletes in South Korea and were registered with the Korean Sport and Olympic Committee. We both hold educational backgrounds in sport and exercise psychology and are committed to the holistic development and wellbeing of athletes. Our experiences as Korean athletes enriched our understanding of the psychological and cultural intricacies of the country's elite sports environment. We acknowledge the potential for unconscious bias inherent in insider research. To mitigate this, we employed a rigorous coding process during data analysis to ensure objective evaluation of the data (Elo et al., 2014). In addition, we consulted with researchers outside the Korean sport culture to broaden our perspective and interpretations.

## 3 Results

Table 2 presents a comprehensive overview of the themes, and their frequencies derived from the analysis of archery performance. Through qualitative analysis, various themes emerged from participants' responses across the different stages of shooting, contributing to a deeper understanding of the ×10 shooting performance. Participants' responses often involved complex and multifaceted experiences, leading to variations in frequency across different phases. Certain phases were naturally highlighted more than others based on personal experiences and what participants found most crucial to their performance, resulting in a higher frequency of reported themes in those phases.

**Table 2:** Content analysis of archery performance themes and frequencies on achieving ×10.

**Phases**	**Movements**	**Main themes**	**Subthemes (frequency)**	**Total frequency (%)**
Preparation	1) Stance; 2) Nocking the arrow	Confidence and positive mindset	Confidence and self-assurance in shooting ×10 (15); Positive thinking (10)	25 (30.9%)
		Loss of self-consciousness	Absence of thoughts (9); Avoidance of overthinking (5); Detachment from score concerns (3)	17 (21%)
		Mastery-oriented goal	Focus on posture (13); Posture correctness (1); Balance maintenance (1)	15 (18.5%)
		Performance-oriented goal	Focus on shooting ×10 (9); Expectation of hitting ×10 (5); Accuracy within 9 points (1)	15 (18.5%)
		Psychological regulation strategies	Sustained focus (6); Visualization (2); Deep breathing (1)	9 (11.1%)
Drawing and anchoring	3) Hooking and gripping; 4) Pre draw; 5) Full draw	Physical techniques and stability	Good balance on both sides (50); Consistency (15); Proper arm positioning (14); No shaking in the arm (8)	87 (55.4%)
		Surrounding environments	Quiet (31); Good weather (11); No wind (7); Light wind (6)	55 (35%)
		Attentional focus	Ignoring surroundings (7); No distracting thoughts (5); Focused attention (3)	15 (9.6%)
Aiming and release	6) Aiming and expansion 7) Release	Wind management strategies	Adjusting shot strength (58); Calculating wind (10); Focusing on 10-point zone (8)	76 (48.4%)
		Natural posture and kinesthetic balance	Natural posture (28); Relaxed body (18); Perfect body balance (9); Steady force transmission (1)	56 (35.7%)
		Confidence and positive outcome expectation	Strong confidence (13); Positive feeling toward 10-point shot (9); Feeling assured (2)	25 (15.9%)
Post-shot	8) Follow-through	Positive emotion elevation	Relief (38); Fun (11); Thrill (7); Pleasure (6); Excitement (6); Coolness (4); Uplifted mood (2)	74 (74%)
		Cognitive affirmation	Confidence from past success (9); Smooth experience (2); Consistency with practice (2); Ease (1)	14 (14%)
		Tension management	Composure maintenance (7); Staying calm (4); Deep breathing (1)	12 (12%)

Phases were categorized based on the sequential movements in archery, from preparation to post-shot follow-through, to provide a comprehensive view of the performance process.

### 3.1 Preparation phase (stance; nocking the arrow stages)

One key theme that emerged within the preparation phase was the importance of *confidence and positive mindset* (30.9%). Korean archers frequently described maintaining a high self-belief and positive outlook during the stance and nocking the arrow stages, reflecting back on past experiences of achieving ×10 scores. Participants emphasized the importance of positive self-talk, with some expressing statements like “Think positively about anything” and “I am confident to shoot a ×10.” Others conveyed a sense of determination through phrases like “I can do it” and “I am confident.” For instance, some participant mentioned,

I always tell myself that I can do it, especially when I am preparing the arrow (Korean archer 31).

I remembered the moment I shot ×10…. I kept telling myself multiple times that I am confident in every short (Korean archer 19).

Another archer also shared the benefits of positive thinking, stating, “Positive thoughts help me stay calm and focused” (Korean archer 2). This highlights the role of confidence and positive mindset in the preparation phase, suggesting that a strong mental approach is crucial for optimal performance.

In addition, a prominent theme related to achieving a state of flow during the preparation phase was characterized by *loss of self-consciousness* (21%), where archers described quieting their minds and minimizing distractions. Korean archers described a mental state characterized by an absence of thoughts, avoidance of overthinking, and detachment from score concerns during the preparation stages. Participants shared experiences of achieving a clear mental state. Some participant shared the experiences:

I tried to clear my mind of any thoughts before shooting (Korean archer 12).

I found that not thinking about anything helped me stay relaxed and in control (Korean archer 37).

Some archers stressed the importance of not overthinking and instead focusing on the task, as one participant stated, “I avoid overthinking about the score and just focus on my posture and breathing” (Korean archer 45). This information indicates that achieving a state of mental clarity and detachment from outcomes might help archers maintain focus and execute their shots effectively.

The findings revealed two distinct themes related to goal orientations during the preparation phase: *mastery-oriented goals* and *performance-oriented goals* (18.5% each). Some archers focusing on *mastery-oriented goals* emphasized maintaining proper form and technical execution. Their statements reflected a focus on internal processes rather than external outcomes, which included concentrating on posture, maintaining correct posture, and ensuring balance:

I just focused on my posture; it helped me maintain balance and shoot the ×10 scores (Korean archer 18).

Maintaining the correct posture was crucial for my performance of getting ×10 (Korean archer 27)

These quotes illustrate how focusing on mastering the technical aspects (e.g., posture and balance) during the preparation is critical to their success in hitting ×10 scores.

In contrast, archers with *performance-oriented goals* expressed a clear focus on the desired outcome (scoring a ×10). Their statements revealed a strong emphasis on achieving a specific result. Common keywords from these archers included phrases like “Aiming at a ×10” and “Going to be a ×10.” This finding indicates that some archers benefited from keeping the desired outcome (×10 score) at the forefront of their minds:

My main focus was on shooting a ×10; I visualize it before every shot. Keeping the target in mind helps me concentrate and align my technique to achieve that perfect score (Korean archer 10).

I expected to hit a ×10, and this expectation drove my focus and shooting. Knowing that I can achieve it if I stay focused motivates me throughout the competition (Korean archer 22).

Some Korean archers' experiences highlight how focusing on performance-oriented goals, such as aiming for and expecting to hit a ×10, can be beneficial for archers. The anticipation and mental visualization of achieving a perfect score appear to enhance their concentration and performance.

Lastly, another critical theme identified within the preparation phase that emerged was the use of *psychological regulation strategies* (11.1%). Some Korean archers described employing maintained focus, visualization, and deep breathing before the shot:

Before the ×10 shot, I visualized the arrow hitting the ×10. The mental imagery helped me calm and confident (Korean archer 14).

Taking a deep breath before each shot helps me relax and clear my mind (Korean archer 5).

These quotes showed how psychological regulation strategies played a vital role in helping archers manage their mental state and achieve ×10 scores.

### 3.2 Drawing and anchoring (hooking and gripping; pre draw; full draw)

In the drawing and anchoring phase, which includes hooking and gripping, pre draw, and full draw, a significant theme that emerged was the importance of *physical techniques and stability* (55.4%). Korean archers frequently underlined maintaining good balance on both sides, consistency, proper arm positioning, and the absence of shaking in the arm during these movements. One of the subthemes was maintaining *good balance on both sides*. Maintaining balance on both sides was seen as crucial for executing a precise shot:

Maintaining balance on both sides was essential for a clean and accurate shot at ×10. I focused on evenly distributing my weight and ensuring my stance was stable (Korean archer 12).

If my balance is off, it affects the entire shot. When look back at the moment I shot ×10, I ensured my weight was centered and my support was firmly stable (Korean archer 17).

Consistency was another important subtheme, with Korean archers highlighting the need for a repeatable and reliable technique. Some archers shared, “Consistency in my draw and release is key, and this helped me to achieve the ×10 consistently” (Korean archer 20). Another archer also described, “I practice the same movements repeatedly to ensure that my draw and release are always consistent. This routine helped me to get the ×10” (Korean archer 22).

Proper arm positioning also played a critical role in the stability and accuracy of the shot. For example, one archer mentioned,

Proper arm positioning, especially during the pre and full draw, ensures that my aim is steady and accurate. When I shot ×10, I paid close attention to my elbow and shoulder alignment (Korean archer 33).

Keeping my arm position correct throughout the draw is crucial. [it] helped [me] maintain control and precision shooting ×10 (Korean archer 14).

Lastly, the absence of shaking in the arm was crucial for some archers in maintaining control over the shot. One participant stated, “It's important that my arm doesn't shake during the draw. I focused on keeping my muscles relaxed yet controlled to maintain stability” (Korean archer 29). Another archer shared a similar experience, saying, “If my arm shakes, it throws off my entire shot. I make a conscious effort to keep my arm steady and my grip firm” (Korean archer 25). The participants' responses revealed how physical techniques and stability are vital for archers in the drawing and anchoring phase. These elements helped them maintain control and accuracy, contributing significantly to their success in achieving ×10 scores.

Another significant theme that emerged within the phase was the impact of *surrounding environments* (35%). Common keywords included “quiet”, “no wind”, “light wind”, “pleasantly cool”, and “good weather” when participants recalled succeeding in achieving a ×10 score. A frequently mentioned factor was a quite environment. Some participants shared the experience:

At that moment, it was really quiet… There was no noise and it helped maintain my concentration (Korean archers 31).

During my best performance, everything around me was silent (Korean archer 1).

Overall good weather, including pleasantly cool temperatures and clear sky, were favored by archers for maintaining comfort and focus during the draw:

I think the cool temperature helped me focus on the game (Korean archer 22).

During the game, cool weather kept me comfortable and helps me concentrate to shoot the ×10 (Korean archer 61).

Clear skies and nice weather contribute to my positive mindset, which is crucial for hitting the ×10 scores (Korean archer 38).

The presence of no wind or light wind was also crucial for maintaining control over the shot. For instance, one archer shared, “Luckily, there was no wind when I shot the ×10…so I just aimed at the ×10 target” (Korean archer 15). Another archer mentioned, “A light breeze was manageable, so I focused on my aim [target]” (Korean archer 61). Not surprisingly, these quotes show that surrounding environmental factors are crucial for archers to maintain their focus and control, enhancing their performance in achieving ×10 scores.

Furthermore, a key theme in this phase was *attentional focus* (9.6%). Korean archers accentuated the importance of ignoring distractions, maintaining focused attention, and having no distracting thought to achieve optimal performance. One archer shared, “I try not to pay attention to the surroundings, It's just me and the target and nothing else matters” (Korean archer 44). Korean archer 50 also explained, “I make sure to clear my mind of any distracting thoughts. It helped me focus entirely on my technique and aim at ×10.” Further, Korean archer 32 noted, “When I'm focused, I don't hear or see anything around me. My attention was solely on hitting the ×10” These quotes revealed that maintaining attentional focus by ignoring distractions and clearing mind of unnecessary thoughts is essential for archers to achieve high levels of performance.

### 3.3 Aiming and release (aiming and expansion; release)

A dominant theme that emerged during the aiming and release phase was *wind management strategies* (48.4%). Korean archers frequently mentioned the importance of adjusting shot strengths, calculating the wind, and focusing on the 10-point zone to maintain accuracy under varying wind conditions:

When the wind picks up, I adjust my shot strength accordingly. It's all about finding the right balance to ensure the arrow stays on course (Korean archer 21).

I always take a moment to calculate the wind before releasing the arrow. Understanding the wind's direction and speed is the key to shoot precisely (Korean archer 43).

Other participants also shared their strategies for dealing with windy weather: “Even in windy conditions, I focus on the 10-point zone. Keeping my aim steady and consistent support me stay on target despite the wind” (Korean archer 36). Korean archer 60 also discussed, “When the wind is strong, I slightly adjust my aim to compensate. It's about making small adjustments to ensure the arrow lands where I want it to.”

Another significant theme was the importance of natural posture and kinesthetic balance (35.7%). Archers commonly mentioned the need for a natural posture, a relaxed body, perfect body balance, and steady force transmission to maintain accuracy and control. One participant pointed out the importance of natural postures, “Maintaining a natural posture, like during practice, felt comfortable and stable while pulling, and helped me shoot more accurate” (Korean archer 25).

The necessity of having a relaxed body was also highlighted by several participants. One archer shared, “Keeping my body relaxed helps me stay calm and focused, which is essential for a precise shot [×10]” (Korean archer 30). Another participant added, “A relaxed body reduced my tension and shoot comfortably” (Korean archer 14). Perfect body balance was another critical aspect mentioned by the archers. One participant explained, “Having perfect balance on both sides was the key to a stable shot of ×10” (Korean archer 20). Similarly, another archer noted, “Balance is everything in archery [The balance] helps to get consistent shots” (Korean archer 59).

The final prominent theme that emerged during the aiming and release phase was the role of *confidence and positive outcome expectation* (15.9%). Korean archers often expressed strong confidence, a feeling of assurance, and a positive outlook toward hitting the 10-point shot, similar to the preparation stage. The participant highlighted the importance of confidence, stating, “Feeling confident is crucial. When I believe in myself, my shots are more precise” (Korean archer 33). Positive feelings toward achieving a 10-point shot were commonly mentioned. One participant noted, “When I have a positive feeling about performance while aiming for the target, I stay motivated and focused” (Korean archer 37). The feeling of assurance also played a significant role. Korean archer 28 archer explained, “Being assured in my skills and technique gives me the stability I need to perform well.”

### 3.4 Post-shot (follow-through)

A prominent theme that emerged during the post-shot, especially in the follow-through stage, was *positive emotion elevation* (74%). Archers frequently described feelings of relief, fun, thrill, pleasure, excitement, coolness, and an uplifted mood after hitting the ×10. Participants expressed, “After shooting a perfect ×10, I felt a wave of relief wash over me. It was like all the pressure just disappeared” (Korean archer 12), “I felt a surge of excitement and pleasure every time I hit the ×10. It was so cool” (Korean archer 31), and “There's a thrilling rush when you see the arrow hit the center. It's an incredible feeling” (Korean archer 19).

*Cognitive affirmation* was another significant theme (14%). Archers often reflected on their confidence stemming from past successes, the smoothness of the experience, consistency with practice, and a general sense of ease. Some participants remarked on the confidence gained from previous achievements:

Each time I hit the ×10, it reinforces my confidence. It reminds me of all the successful shots I have made in the past (Korean archer 23).

Every perfect shot builds my confidence, reminding me that I can replicate my success (Korean archer 8).

These quotes indicate that successful experiences reinforce confidence, contributing to future success.

The final theme that emerged was *tension management* (12%). Archers emphasized the importance of maintaining composure, staying calm, and using deep breathing techniques to manage tension after the shot. One archer shared, “It is crucial to keep my composure after shooting. Staying calm helps me prepare for the next shot” (Korean archer 3). Another participant highlighted the role of deep breathing, saying, “Deep breathing helps me manage any lingering tension and stay focused” (Korean archer 61). These themes illustrate how Korean archers navigate their emotional and cognitive states during the post-shot follow-through phase, contributing to their overall success in achieving consistent high performance.

## 4 Discussion

This study provides comprehensive insights into the detailed strategies, techniques, and coping skills for external factors that influence the ×10 performance among Korean archers, based on the sequences of archery movements.

This study identified various mindsets and strategies that Korean archers used during the preparation stage to achieve ×10 scores. Consistent with existing research on successful athletes' mental states before competition, including confidence, optimism, and self-talk (McNeil et al., 2023; Norsworthy et al., 2018), our findings underscore the importance of a confidence and optimistic mindset (e.g., positive anticipations about performance) and the experience of flow (e.g., a state of complete immersion and focus) in achieving peak performance. These results suggest that archers who engage in positive self-talk or enter a state of deep concentration are more likely to score ×10.

We also discovered that Korean archers adopt goal-oriented approaches, either mastery- or performance-based, in their preparation phase. Drawing from the foundational principles of achievement goal model (Ames, 1995; Nicholls, 1989), mastery-oriented goals emphasize personal skill development and task mastery whereas performance-oriented goals focus on external validation through winning or success. The debate continues as to whether one orientation clearly leads to better outcomes in sports or if it is more effective to consider both orientations in tandem (Rottensteiner et al., 2015; Tenenbaum et al., 1999). Mastery-oriented goals have been prevalently observed among athletes competing at higher levels (Ong, 2019) and are associated with greater motivational outcomes (Jõesaar and Hein, 2011; Ntoumanis, 2001) when compared to performance-oriented goals. Contrary to previous studies, our research indicated that Korean archers employed both mastery and performance-oriented strategies. It is important to consider, however, that while some archers expressed performance-driven goals, their main focus was on hitting the ×10 rather than winning competitions. This aligns with research on the effectiveness of process goals, which emphasizes the specific actions and techniques needed for success (Williamson et al., 2022). The systematic and meta-analysis conducted by Williamson et al. (2022) showed that process goals had a greater impact on performance compared to outcome goals. By focusing on these process goals, archers can maintain consistent performance, a critical factor in high-pressure competitions.

In addition, we also observed that some Korean archers utilized attention management and psychological regulation techniques. These skills could be particularly crucial in archery, a sport that demands precise focus and concentration, and are vital for managing the intense pressure (Kim et al., 2021; Medeiros Filho et al., 2008). This discovery resonates with and builds upon existing literature, underscoring the significance of attentional abilities (Vrbik et al., 2021) as well as psychological regulation techniques (i.e., breath control and imagery) in enhancing athletic performance (Kim and Kim, 2020; Laborde et al., 2022; Salleh et al., 2020).

In the critical execution stages, from drawing to releasing shooting of Korean archery, both external and internal factors converge to influence the likelihood of achieving ×10 scores. This study highlighted the crucial importance of balance and strength in archery, aligning with previous findings that identify these as key factors in the sport (Kim et al., 2015). The integration of balance and strength into training regimens could significantly enhance performance. Furthermore, as we expected in the field of archery, the study indicated a significant influence of external environmental factors (e.g., wind conditions, acoustic disturbance, climatic variability) on Korean archers' performance, highlighting their adept management of these elements. These findings are consistent with previous research emphasizing the importance of adapting to dynamic environmental conditions in sports (Lu et al., 2021; Vrbik et al., 2021).

Unlike many sports, archery requires a high level of concentration and precision, with external factors like wind and noise potentially disrupting performance (Kim et al., 2021; Lu et al., 2021). To adapt to such environmental disturbances, Korean archers skillfully adjusted their techniques and strategies in response to varying conditions, demonstrating a blend of physical strength and mental resilience. Holistic approaches, which encompass mental agility (i.e., focusing on posture or the target and managing external distractions) and environmental awareness (i.e., understanding wind speed and direction) were seamlessly integrated with physical skills to precisely achieve the ×10 score among the Korean archers. These insights have the potential to guide the creation of holistic training programs. Such programs would extend beyond mere technical skill enhancement, incorporating strategies for adaptive responses to environmental variables and training that sharpens sensory acuity (Lu et al., 2021; Vrbik et al., 2021).

We observed the mental and emotional states experienced by the archers when achieving ×10 because comprehending these aspects is crucial for sustaining high-level performance. The Korean archers displayed, as expected, positive emotional states (i.e., excitement, pleasant, confidence, and calmness) which are akin to the emotional responses of athletes in other sports when they achieve peak performance (Cooper et al., 2021). However, this study also revealed that some Korean archers actively sought to regulate their emotions and set subsequent goals. This approach suggests a strategic and proactive stance toward emotional management, indicating that these archers do not merely experience emotions passively but rather actively work to harness and channel them constructively.

Our study intriguingly found an absence of references to negative thoughts, anxiety, or anger during ×10 shooting. This finding contrasts with the emotional experiences reported in dynamic sports like running (Lane et al., 2011) and contact sports (e.g., American football, basketball, ice hockey; Campo et al., 2012). This distinction may be attributed to the static nature of archery, which emphasizes precision and steadiness. These findings imply that fostering positive mindsets or achieving a state of flow can lead to successful outcomes in sports-related shooting techniques (Haywood, 2005; Kim et al., 2021; Salleh et al., 2020). Techniques observed in the archers, such as deep breathing, visualization, and self-talk, were utilized to manage excitement and mitigate negative emotions. These strategies appear to be key in maintaining a focused and serene state, which is essential for achieving ×10 scores.

### 4.1 Limitation

It is crucial to acknowledge certain limitations and propose directions for future research. The investigation focuses on the specific experiences of Korean archers in achieving ×10 scores, which are deeply rooted in a unique sporting context. This specialized focus may limit the broader applicability of the findings. While the research illuminates aspects of the high performance in Korean archery, it is essential to recognize that athletes from various cultural backgrounds might adopt different approaches in developing mental strategies for peak performance (Broch and Kristiansen, 2014). Thus, future studies could extend to archers with diverse cultural perspectives. Exploring the potential for the systematic cultivation of these mental strategies among younger or less experienced archers also could also offer valuable insights for enhancing performance across different levels of archery expertise.

## 5 Conclusion

This study aimed to uncover the key factors behind the outstanding performance of Korean archers, with a particular focus on their ability to achieve ×10 scores. Our research provided a detailed map of their strategies, techniques, and coping skills for managing external factors, all organized around the sequential phases of archery movements. This holistic approach can help archers develop the tools needed to handle the psychological demands of high-stakes competitions and maintain consistent performance under pressure. The insights from this study offer practical applications for archers, coaches, and sport psychologists working with archers, guiding the development of interventions to enhance mental strategies, physical techniques, and environmental coping skills, thereby improving performance outcomes in archery sports.

## Data Availability

The study datasets are unavailable publicly due to privacy and ethical concerns but may be requested from the corresponding author under institutional and ethical guidelines.
